# Supplementary material for: Splice-Junction-Based Mapping of Alternative Isoforms in the Human Proteome
Source: Cell Rep. Author manuscript; Available in PMC 2020 Jan 15. (PMC6961840; doi:10.1016/j.celrep.2019.11.026)
Supplement: 2 [file NIHMS1546469-supplement-2.zip › DF1/PXD009737/TestisGluC/TestisGluC_9_ADD1_PSCITNCLVTSPPPTTAPHKE.pdf]

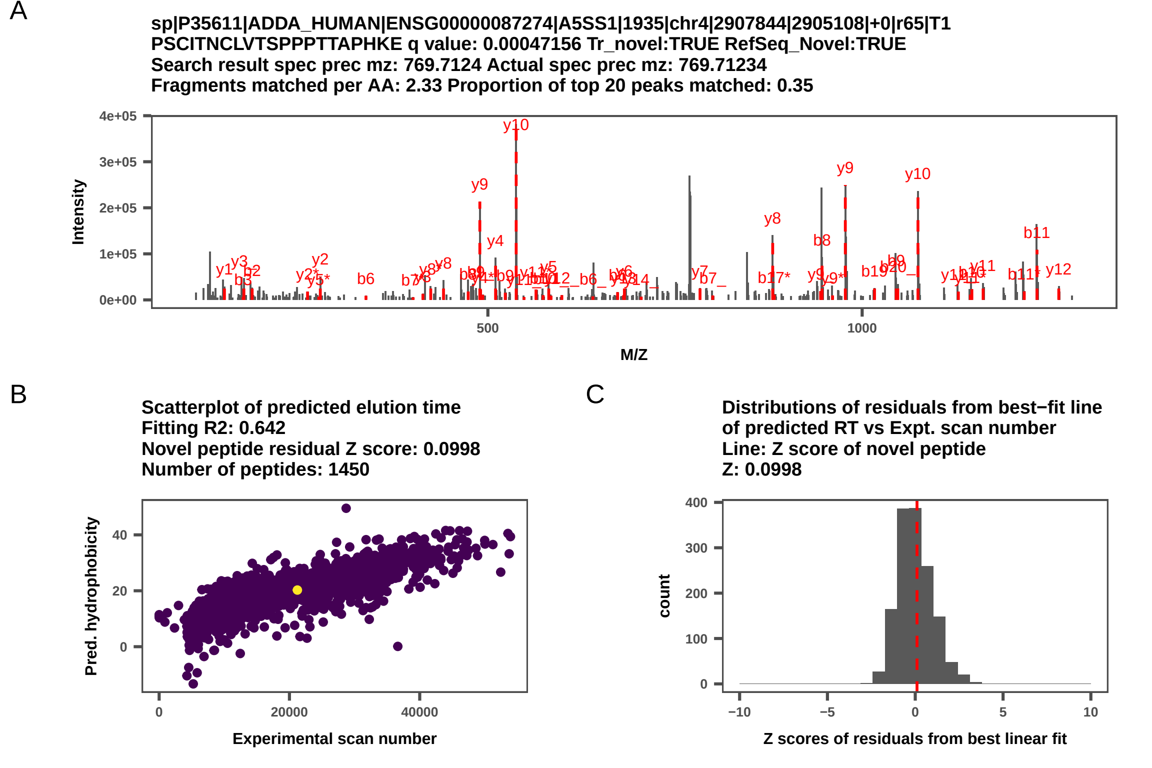
A
sp|P35611|ADDA_HUMAN|ENSG00000087274|A5SS1|1935|chr4|2907844|2905108|+0|r65|T1
PSCITNCLVTSPPPTTAPHKE q value: 0.00047156 Tr_novel:TRUE RefSeq_Novel:TRUE
Search result spec prec mz: 769.7124 Actual spec prec mz: 769.71234
Fragments matched per AA: 2.33 Proportion of top 20 peaks matched: 0.35
Intensity
4e+05
3e+05
2e+05
1e+05
0e+00
y10
y9
y4
y8
b8
y9
y10
b11
y12
b6
500
1000
M/Z
B
Scatterplot of predicted elution time
Fitting R2: 0.642
Novel peptide residual Z score: 0.0998
Number of peptides: 1450
Pred. hydrophobicity
40
20
0
0
20000
40000
Experimental scan number
C
Distributions of residuals from best−fit line
of predicted RT vs Expt. scan number
Line: Z score of novel peptide
Z: 0.0998
count
400
300
200
100
0
−10
−5
0
5
10
Z scores of residuals from best linear fit
